# Production of medium-chain-length polyhydroxyalkanoates by sequential feeding of xylose and octanoic acid in engineered *Pseudomonas putida* KT2440

**DOI:** 10.1186/1472-6750-12-53

**Published:** 2012-08-22

**Authors:** Sylvaine Le Meur, Manfred Zinn, Thomas Egli, Linda Thöny-Meyer, Qun Ren

**Affiliations:** 1 Laboratory for Biomaterials, Swiss Federal Laboratories for Materials Science and Technology (Empa), Lerchenfeldstrasse 5, St. Gallen, CH-9014, Switzerland; 2 Biotechnology, HES-SO Valais Wallis, Rue du Rawyl 64, P.O.B. 2134, Sion, CH-1950, Switzerland; 3 Environmental Microbiology, Swiss Federal Institute of Aquatic Science and Technology (Eawag), Überlandstrasse 133, P.O. Box 610, Dübendorf, CH-8600, Switzerland

**Keywords:** mcl-PHA, Xylose, Octanoic acid, *Pseudomonas putida* KT2440, Sequential-feeding, Tailor-made PHA

## Abstract

**Background:**

*Pseudomonas putida* KT2440 is able to synthesize large amounts of medium-chain-length polyhydroxyalkanoates (mcl-PHAs). To reduce the substrate cost, which represents nearly 50% of the total PHA production cost, xylose, a hemicellulose derivate, was tested as the growth carbon source in an engineered *P. putida* KT2440 strain.

**Results:**

The genes encoding xylose isomerase (XylA) and xylulokinase (XylB) from *Escherichia coli* W3110 were introduced into *P. putida* KT2440. The recombinant KT2440 exhibited a XylA activity of 1.47 U and a XylB activity of 0.97 U when grown on a defined medium supplemented with xylose. The cells reached a maximum specific growth rate of 0.24 h^-1^ and a final cell dry weight (CDW) of 2.5 g L^-1^ with a maximal yield of 0.5 g CDW g^-1^ xylose. Since no mcl-PHA was accumulated from xylose, mcl-PHA production can be controlled by the addition of fatty acids leading to tailor-made PHA compositions. Sequential feeding strategy was applied using xylose as the growth substrate and octanoic acid as the precursor for mcl-PHA production. In this way, up to 20% w w^-1^ of mcl-PHA was obtained. A yield of 0.37 g mcl-PHA per g octanoic acid was achieved under the employed conditions.

**Conclusions:**

Sequential feeding of relatively cheap carbohydrates and expensive fatty acids is a practical way to achieve more cost-effective mcl-PHA production. This study is the first reported attempt to produce mcl-PHA by using xylose as the growth substrate. Further process optimizations to achieve higher cell density and higher productivity of mcl-PHA should be investigated. These scientific exercises will undoubtedly contribute to the economic feasibility of mcl-PHA production from renewable feedstock.

## Background

Polyhydroxyalkanoates (PHAs) are bacterial storage compounds produced widely by many microorganisms under nutrient limited growth conditions such as a nitrogen, phosphorous or oxygen starvation and when an excess of carbon source is present [1,2]. PHAs gained particular interest because they were shown to be biodegradable and biocompatible (see review [3]). Based on the chain length of the fatty acid monomers, PHAs can be classified into three categories: short-chain-length (scl) PHAs with 3 to 5 carbon atoms, medium-chain-length (mcl) PHAs with 6 to 14 carbon atoms and long-chain-length (lcl) PHAs with more than 14 carbon atoms [4]. The difference in length and/or chemical structure of the alkyl side chain of the PHAs influences the material properties of the polymers to a great extent. The production of tailor-made mcl-PHAs enables to obtain the desired material properties using the appropriate fatty acid precursor. PHAs have been considered as an attractive ecofriendly alternative to petrochemical polymers. However, the much higher production cost compared with conventional petrochemical derived polymers has limited their widespread use.

Much effort has been devoted to reduce the price of PHAs by developing better bacterial strains, more efficient fermentation and/or more economical recovery processes [6-11]. It has been shown that the cost of raw materials (mainly the carbon source) contributes most significantly to the overall production cost of PHAs (up to 50% of the total production cost) [12]. The use of two kinds of carbon sources can be an attractive approach to reduce cost: the first carbon substrate is used for cell growth to obtain biomass, while the second one (which may be more expensive) allows the synthesis of PHA. The substrate for bacterial growth should be inexpensive and abundant. Xylose is second only to glucose in natural abundance [13]. Thus, it is a promising candidate substrate for bacterial growth.

D-Xylose is the dominant building unit of the hemicelluloses in plants of all species of the *Gramineae*. Hemicellulose, the third most abundant polymer in nature, can be easily hydrolyzed into fermentable sugars by either chemical or enzymatic hydrolysis [14]. In some plants, xylan comprises up to 40% of the total dry material. Annually, 60 billion tons of hemicelluloses are produced and remain almost completely unused [15]. It has been reported that the hemicellulose hydrolysate including xylose can be used by *Candida blankii* for efficient protein production [16]. There are also reports that poly(3-hydroxybutyrate) (PHB) could be synthesized from xylose in *Pseudomonas pseudoflava* and *P. cepacia* up to 22% (w w ^-1^) and 50% (w w ^-1^), respectively [17-19]. Furthermore, *Escherichia coli* harboring PHA synthesis genes of *Ralstonia eutropha* was reported to be able to accumulate PHB from xylose up to 74% w w ^-1^ with a yield of 0.226 g PHB per g xylose [20].

Up to now, no report has been published on the production of mcl-PHA by using xylose. Since mcl-PHAs offer different material properties compared to scl-PHAs, it would be interesting to investigate whether mcl-PHAs can be obtained from xylose. *Pseudomonas putida* KT2440, whose genome sequence is available ( http://www.ncbi.nlm.nih.gov), is one of the best-characterized pseudomonads for mcl-PHA production [21]. It is able to synthesize and accumulate large amounts (up to 75% w w ^-1^) of mcl-PHAs [22], but can only ferment a narrow range of sugars, in which xylose is not included. It has been shown that an engineered strain of *P. putida* S12 can utilize D-xylose and L-arabinose [23]. Introducing *xylA* (encoding xylose isomerase) and *xylB* (encoding xylulokinase) from *E. coli* into *P. putida* S12 enabled the latter to utilize xylose as the sole carbon source.

In this study, the possibility of using xylose as a growth carbon source and octanoic acid as mcl-PHA precusor in the controlled production of mcl-PHA by recombinant *P. putida* KT2440 was examined. The *xylAB* genes from *E. coli* W3110 were cloned into *P. putida* KT2440 and the obtained recombinant was studied for its ability to grow on xylose. For mcl-PHA production a sequential feeding strategy of using xylose and fatty acids was applied.

## Results

### Cloning and expression of the *xylA* and *xylB* genes encoding xylose isomerase and xylulokinase

To clone the *xylAB* genes, which are organized in an operon, of *E. coli* W3110, DNA primers PFXylA1 and PRXylB were designed based on the genomic sequence of *E. coli* W3110 ( http://www.ncbi.nlm.nih.gov). These primers were used to amplify the *xylAB* fragment by PCR with W3110 chromosomal DNA as the template, leading to a 2.85 kb DNA product (*xylAB*). The PCR product was inserted into the shuttle vector pVLT33 as described in Methods, resulting in pSLM1 plasmid. *P. putida* KT2440 (pSLM1) was grown on E2 minimal medium with either 10 g L^-1^ xylose or glucose, leading to a C/N ratio of 17 g g ^-1^. *P. putida* KT2440 (pVLT33) was used as a control.

**Figure 1:**
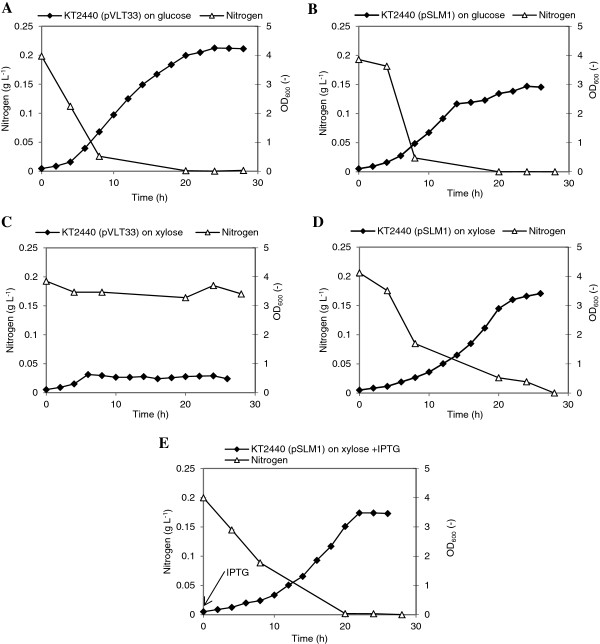
**Growth of the *P. putida *KT2440 recombinants in shake flasks on E2 minimal medium containing 10 g L**^**-1**^**glucose or xylose as the sole carbon source.** The arrow represents the addition of IPTG. The experiments were performed in replicate flasks. Data points are the averages of the results of duplicate measurements.

When grown on glucose, KT2440 (pVLT33) exhibited a higher maximal specific growth rate (0.32 h^-1^) than KT2440 (pSLM1) (0.26 h^-1^) (Figure 1A &1B). Due to nitrogen limitation both KT2440 (pVLT33) and KT2440 (pSLM1) exhibited reduced growth rate after 14 h, with the latter having a dramatic reduction (Figure 1B). The difference in the degree of the reduction in growth rate could be partially caused by the different PHA content in KT2440 (pVLT33) (about 20% w w^-1^) and KT2440 (pSLM1) (about 1% w w^-1^). The different PHA content was also reflexed by the different maximum OD_600_ values reached by KT2440 (pVLT33) (4.28) and KT2440 (pSLM1) (2.94) (Figure 1A &1B). When xylose was used as the sole carbon source, KT2440 (pVLT33) was not able to grow during the entire test period (Figure 1C), whereas the recombinant KT2440 (pSLM1) exhibited a typical bacterial growth curve (Figure 1D & 1E). The maximum specific growth rate of the IPTG-induced recombinant KT2440 (pSLM1) culture was similar to the culture without induction, of μ = 0.23 h^-1^ and μ = 0.21 h^-1^, respectively. This demonstrates that the expression of *xylAB* from pSLM1 is not tightly regulated and *xylAB* can be expressed even without the induction by IPTG. The recombinant KT2440 (pSLM1) reached a maximal OD_600_ value of 3.66 on xylose. In all experiments the carbon substrate (either glucose or xylose) was not totally consumed at the end of the cultivation, around 4 g L^-1^ was left over in the culture broth.

These results suggest that the cloned *xylAB* from *E. coli* are functionally expressed in *P. putida* KT2440, and *xylAB* alone are sufficient to allow the growth of KT2440 on xylose. To have better controlled growth, further experiments were performed in bioreactors.

### Growth of *P. putida* KT2440 (pSLM1) on xylose in the bioreactor

**Figure 2:**
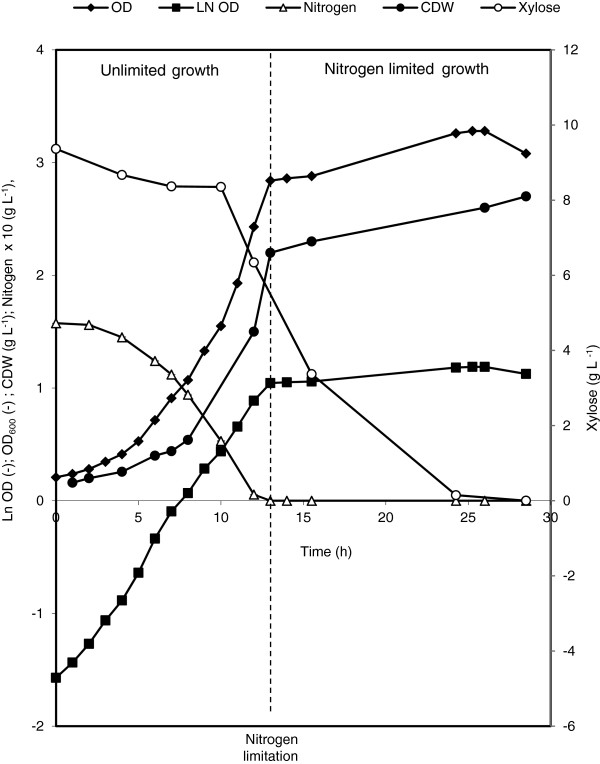
**Growth of *P. putida *KT2440 (pSLM1) in E2 minimal medium with 10 g L**^**-1 **^**xylose in a 3.7 L bioreactor.** Data points are the averages of the results of duplicate measurements.

*P. putida* KT2440 (pSLM1) was grown on E2 medium with 10 g L^-1^ xylose in a 3.7 L laboratory bioreactor. Figure 2 shows that the KT2440 (pSLM1) cells utilized xylose as the sole carbon source with a maximum specific growth rate of 0.24 h^-1^. The growth stopped due to nitrogen limitation after 13 h of cultivation. Afterwards the biomass increased only slightly from 2.2 g L^-1^ to maximum 2.7 g L^-1^ at 28 h. Even though nitrogen-limitation was reached, xylose was further consumed and finally only a tiny amount was left in the medium (Figure 2). The biomass yield from xylose was about 0.5 g g^-1^ upon nitrogen limitation (13 h of cultivation), and about 0.27 g g^-1^ upon the xylose depletion. The consumed xylose after nitrogen completion may have been used for cell maintenance and/or by-products such as acetic acid. Indeed, large amounts of acetic acid were detected from the beginning of the growth, in the range of several hundred milligrams per liter. The culture was also assayed for PHA content at different time points. Only trace amounts (up to 0.3% w w^-1^) of PHA were detected using xylose as the sole carbon source, which enables to use xylose as growth substrate for the production of tailor-made mcl-PHAs.

To confirm the enzymatic activities of XylA and XylB, cells were harvested at the early exponential growth phase (OD_600_ of about 0.5). Samples without substrates and samples without cell-free extracts were used as negative controls, while *E. coli* W3110 cells grown on E2 with xylose were used as the positive control. The specific activities of xylose isomerase and xylulokinase measured in *P. putida* KT2440 (pSLM1) were 1.47 U and 0.97 U, respectively, whereas no significant activites were observed in the negative controls (Table 1). The activities obtained here were in the same range as those found in wild-type *E. coli* W3110 (Table 1). These results confirmed that both XylA and XylB were active in *P. putida* KT2440. The enzymatic activities of XylA and XylB were also found for the non-induced cultures, thus, induction by IPTG is not needed for the expression of *xylA* and *xylB* genes and was therefore omitted in the following experiments.

**Table 1:** **Enzymatic activities of XylA and XylB in *P. putida *KT2440 (pSLM1)**

	**Negative control**		**Sample**	
	**No substrate**	**No cells**	***E. coli *W3110**	***P. putida *(pSLM1)**
XylA act. (*U*)	0.07	0.33	1.79	1.47
XylB act. (*U*)	0.26	0.12	1.39	0.97

***U***: One unit is defined as 1 μmole of consumed NADH min ^-1^.

### PHA production in KT2440 (pSLM1) by sequential-feeding of xylose and fatty acid

Nitrogen limitation is known to promote PHA accumulation [1]. It has been demonstrated that nitrogen limitation can lead to a strong induction of *phaG* encoding a transacylase, resulting in mcl-PHA accumulation from carbohydrate in *P. putida* KT2440 [24]. Thus, in the experiments performed in mini-reactors the amount of nitrogen present in E2 medium was decreased to 20% (namely 0.2NE2 medium) to obtain the best conditions for mcl-PHA accumulation. As expected, KT2440 (pVLT33) did not show any growth on xylose (Table 2, culture A), similar as observed in Figure 1. KT2440 (pSLM1) exhibited a maximal specific growth rate of 0.24 h^-1^ on xylose with a maximum OD_600_ of 0.99 (Table 2, culture B). No PHA was detected for KT2440 (pSLM1) on xylose.

**Table 2:** PHA production in batch and fed-batch fermentations

**Entry**	**Strains**	**Fermentation type**	**Carbon source**		**Feeding type/duration**	**Feeding phase**	**Flow**	**Total amount of fed octanoic acid**	**OD**_**600**_	**μ**_**max**_**(h**^**-1**^**)**	**PHA content (% w w**^**-1)**^
			**growth substrate**	**PHA precusor**							
A	KT2440 (pVLT33)	Batch	xylose	-	-	-	-		no growth	-	-
B	KT2440 (pSLM1)	Batch	xylose	-	-	-	-		0.99	0.24	0.3
C	KT2440 (pSLM1)	Batch	octanoic acid	-	-	-	-	1.44 g L^-1^	2.77	0.35	21.0
D	KT2440 (pSLM1)	Fed-Batch	xylose	octanoic acid	linear/12 h	mid exp. ^(1)^	0.072 g L^-1^ h^-1^	0.87 g L^-1^	1.48	0.24	12.1
E	KT2440 (pSLM1)	Fed-Batch	xylose	octanoic acid	linear/8 h	end exp. ^(2)^	0.072 g L^-1^ h^-1^	0.58 g L^-1^	1.52	0.25	16.2
F	KT2440 (pSLM1)	Fed-Batch	xylose	octanoic acid	linear/4 h	end exp. ^(2)^	0.288 g L^-1^ h^-1^	1.15 g L^-1^	1.4	0.25	20
G	KT2440 (pSLM1)	Batch	xylose + octanoic acid	octanoic acid	-	-	-	1.44 g L^-1^	2.13	0.27	28.7

The cells were grown in 0.2NE2 minimal media supplemented with different carbon sources in 1 L mini-bioreactors with a working volume of 800 mL. Samples were taken regularly to measure growth, nitrogen concentration, carbon source present in the medium and PHA contents. Data points are the averages of duplicate measurements.

^(1)^: Feeding started at the middle of exponential batch phase, OD_600_ of 0.5.

^(2)^: Feeding started at the end of exponential batch phase, OD_600_ of 1.

To test whether the recombinant is able to accumulate mcl-PHA from related carbon source (e. g. octanoic acid), KT2440 (pSLM1) was pre-cultured in E2 minimal medium with xylose as the sole carbon source and then transferred into a bioreactor containing 0.2NE2 medium with 10 mM octanoic acid as the sole carbon source (Table 2, culture C). Samples were taken regularly and the cellular PHA content was determined. It was found that KT2440 (pSLM1) was able to accumulate mcl-PHA to 21% (w w^-1^) after 33 h of cultivation.

The above results demonstrate that the recombinant strain KT2440 (pSLM1) kept the ability to synthesize PHA from fatty acids, but could not do so from xylose. This allows sequential feeding of xylose and fatty acids to obtain tailor-made mcl-PHA biosynthesis, namely, first using an inexpensive carbon source for cell growth and then adding the appropriate mcl-PHA precursor to allow the polymer accumulation. The sequential feeding strategy enables production of a tailor-made mcl-PHA according to the supplied fatty acid. Octanoic acid was tested here as mcl-PHA precursor in order to obtain poly(3-hydroxyoctanoate) accumulation. We first investigated the influence of linear feeding of octanoic acid at different growth stages on PHA synthesis. Four minireactors in parallel were inoculated using the same *P. putida* KT2440 (pSLM1) preculture grown in E2 medium with 1.8 g L^-1^ xylose. In cultures D and E linear feeding of 0.072 g L^-1^ h^-1^ of octanoic acid was initiated at OD_600_ of 0.5 (8 h of batch cultivation) and 1.0 (10 h of batch cultivation), respectively. Both cultures showed a similar maximum specific growth rate of 0.24 h^-1^ (Table 2). The nitrogen limitation was reached after 11 h and xylose was depleted after 12 h for both cultures. The maximum accumulation of PHA was found to be 12.1% w w^-1^ and 16.2% w w^-1^ for D and E, respectively, after about 20 h of cultivation (Table 2). The obtained results suggested that PHA can be produced in *P. putida* KT2440 (pSLM1) by sequential feeding of xylose and fatty acids. The exponential phase ended at OD_600_ of 1.0 due to nitrogen limitation. Linear feeding at the end of the exponential growth phase in the batch (OD_600_ of 1.0) gave a better yield of PHA compared to that at the mid-exponential growth phase (OD_600_ of 0.5). This lower yield obtained in the latter can be explained by consumption of octanoic acid for growth rather than for PHA accumulation during the mid-exponential phase.

**Figure 3:**
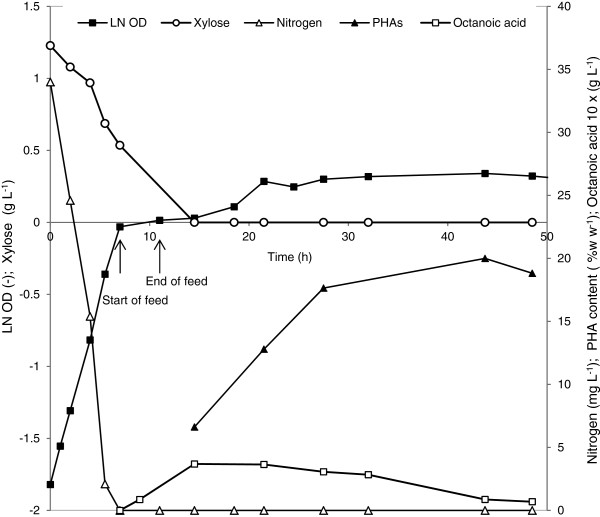
**Fed-batch experiment of *P. putida *KT2440 (pSLM1) grown on 0.2NE2 medium containing 1.8 g L**^**-1 **^**xylose with linear feeding of 0.288 g L**^**-1**^**h**^**-1**^** octanoic acid at OD**_**600 **_**of 1 for 4 h.** Nitrogen (∆), octanoic acid (□) and xylose (○) concentrations were measured. The logarithmic growth is represented by filled squares (■) and the PHA accumulation by filled triangles (▲). Data points are the averages of the results of duplicate measurements.

To increase PHA accumulation, the feeding rate was increased from 0.072 g L^-1^ h^-1^ to 0.288 g L^-1^ h^-1^ of octanoic acid. Culture F was grown on 0.2NE2 with 1.8 g L^-1^ xylose and feeding started at the end of the exponential growth phase with a feeding rate of 0.288 g L^-1^ h^-1^ of octanoic acid. For comparison, culture G was supplemented with 1.8 g L^-1^ xylose and 1.44 g L^-1^ octanoic acid from the beginning on (batch culture on mixed carbon sources). Using culture F as an example, Figure 3 represents a typical behavior of cells regarding growth and PHA synthesis. After 7 h and 14.5 h of cultivation, nitrogen and xylose were depleted, respectively. When cells entered the nitrogen limitation phase a linear feeding of octanoic acid was started for 4 h and then stopped. PHA synthesis was detected after 2 h of feeding and increased linearly to 17.6% w w^-1^ during the following 14 h. Only a slight increase of PHA content to about 20% w w^-1^ was found after further incubation up to 43 h. The concentration of octanoic acid measured in the culture increased with feeding time, after 7.5 h from the beginning of the feeding about 0.369 g L^-1^ octanoic acid was detected; afterwards it continuously decreased. At the end of the cultivation, after 48.5 h, the remaining octanoic acid was only 0.069 g L^-1^. A maximal yield of mcl-PHA from octanoic acid of 0.37 g mcl-PHA g^-1^ octanoic acid was obtained by considering the increase of PHA from 6.6% w w^-1^ to 20% w w^-1^ in relation to the consumption of octanoic acid from 0.369 g L^-1^ to 0.086 g L^-1^.

GC analysis revealed that the main monomer component of the synthesized PHA was 3-hydroxyoctanoate (87% w w^-1^) and no 3-hydroxydecanoate was detected. These results confirm that the detected PHA is mainly from octanoic acid. Since the main monomer unit of PHA produced from carbohydrates is 3-hydroxydecanoate in KT2440 [25,26], it is very unlikely that xylose was used for PHA synthesis. The sequential feeding strategy using xylose as growth substrate and octanoic acid as mcl-PHA precursor enabled production of a controlled mcl-PHA production.

## Discussion

### Growth of KT2440 on xylose

**Figure 4:**
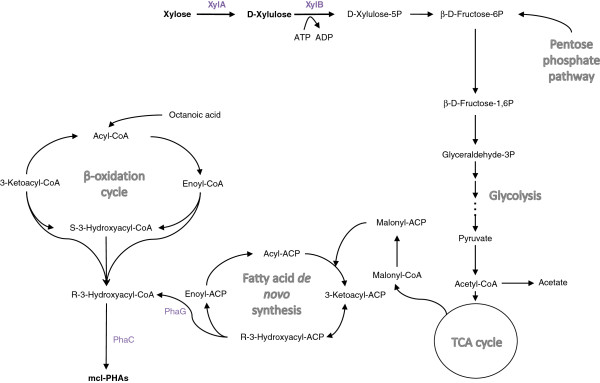
**Hypothetical pathway for mcl-PHA accumulation from xylose in *P. putida*.** Enhanced arrows: steps absent in wild-type *P. putida* strains; XylA: xylose isomerase; XylB: xylulokinase; PhaG: 3- hydroxyacyl-ACP:CoA transferase; PhaC: PHA polymerase.

A recombinant *P. putida* KT2440 strain was constructed that could efficiently utilize xylose. The introduction of xylose isomerase (XylA) and xylulokinase (XylB) was essential and sufficient for the utilization of xylose and a growth rate of 0.24 h^-1^ was routinely obtained. Previously, it has been reported that a so called “laboratory evolution” was necessary to improve the growth rate of *P. putida* S12 (*xylAB*) on xylose from 0.01 h^-1^ to 0.35 h^-1^ and to obtain a yield of 0.52 g CDW per g xylose [23]. The laboratory evolution is an adoption process by growing the cells consecutively in a fresh medium containing the unfavorable carbon source. The “laboratory evolution” was not needed for the KT2440 recombinant to grow on xylose. This difference could be attributed to the different physiological background/metabolic fluxes of KT2440 and S12. It has been reported that in *P. putida* a complete pentose phosphate pathway is present [23,27] (Figure 4) as well as the key enzymes for mcl-PHA accumulation [28]. Our study demonstrated that the enzymes responsible for converting xylose to the entry intermediate xylulose-5-phosphate of PP pathway are missing in *P. putida*. By introducing the relevant enzymes XylA and XylB, *P. putida* KT2440 was able to utilize xylose.

In addition, the recombinant *P. putida* KT2440 appeared to have an efficient xylose uptake system. Similarly, *P. putida* S12 carrying D-xylonate dehydratase has been reported to grow on xylose without expressing any xylose transporter [29]. Since pentose and hexose transporters have been shown to be promiscuous [30], it is possible that xylose uptake can be accomplished by glucose uptake systems in strain KT2440 (*xylAB*). Many bacteria also possess non-specific transporters. Indeed, many sugars are transported into *E. coli* by phosphoenolpyruvate-dependent phosphotransferase systems (PTS) like glucose, mannose, fructose, and N-acetylglucosamine [31]. In this study, no specific xylose transporters such as XylE or XylFGH were needed for growth of KT2440 (*xylAB*) on xylose. Thus, it is also possible that xylose entered the cell through the PTS system present in *P. putida* in a similar way as reported for fructose [32]. Xylose, after uptake into the cell, is isomerized by xylose isomerase to xylulose, which is then converted by xylulokinase to xylulose 5-phosphate. This phosphorylated derivate is then catabolized by the pentose phosphate pathway. In comparison to growth on glucose, the growth of *P. putida* KT2440 (*xylAB*) on xylose exhibited a similar specific growth rate of 0.24 h^-1^ (Figure 1). This demonstrated that the uptake and the catabolic rate of xylose by the recombinant *P. putida* KT2440 (pSLM1) is in the same range as that of glucose.

### PHA production by sequential feeding

The biosynthesis of mcl-PHA is mainly studied for fluorescent pseudomonads, e.g. *P. putida* KT2440. Strain KT2440 is characterized by a wide metabolic and physiologic versatility and is able to accumulate mcl-PHA from glucose [33]. In this study, we demonstrated that mcl-PHA biosynthesis on xylose does not occur when *xylA* and *xylB* are expressed even under nitrogen limitation, perhaps because the expression of *xylAB* channels the metabolic flux to central metabolism such as TCA cycle for cell maintenance or/and to production of side products like acetate, rather than to PHA synthesis (Figure 4).

Up to now, there has been no report on mcl-PHA production by using xylose as the growth substrate. Substrate cost make up a large proportion of the total production cost of PHA. Fatty acids are generally much more expensive than lignocellulose hydrolysates (such as xylose) and often toxic to the cells at relatively low concentrations and, for some of them, do not support fast growth rates. Xylose is in a similar price range like cane molasses and half the price of glucose [34], consequently, sequential-feeding strategies are a valid option to reduce the production cost [35,36]. Sequential-feeding consists of using on one hand cheap carbohydrates for achieving a large biomass and on the other hand fatty acids as mcl-PHA precursors to produce tailor-made mcl-PHAs.

In this study, xylose was used for cell growth in the first step, and then octanoate was supplied to synthesize mcl-PHA in the second step under nitrogen limitation. This sequential feeding process allowed a tailor-made mcl-PHA accumulation of up to 20% (w w^-1^) under not-yet-optimized conditions. When 1.44 g L^-1^ octanoate was employed alone for growth and PHA production (Table 2, entry C), lower PHA content (about 21% w w^-1^) was obtained than that from using both xylose and 1.44 g L^-1^ octanoate (Table 2, entry G, about 28.7% w w^-1^), even though the growth rate and the final cell density reached in entry C were higher than those in entry G. These results suggest that xylose is not a substrate as good as octanoate for growth of KT2440, however, it can facilitate the PHA production by being a substrate for growth and allowing only octanoate to be converted to PHA.

In this study, *P. putida* KT2440 (pSLM1) showed a biomass yield from xylose at 0.50 g g^-1^, which is similar to what has been previously reported 0.52 g g^-1^ for *P. putida* S12 (*xylAB*) [23]. Previously, Kim and co-workers used a sequential feeding strategy to maximize the PHA production in *P. putida* using glucose as growth substrate and then octanoic acid for PHA accumulation [37]. A yield of 0.4 g mcl-PHA g^-1^ octanoic acid was reached [37], similar to the yield of 0.37 g mcl-PHA g^-1^ octanoic acid obtained in this study by sequential feeding of xylose and octanoic acid. However, it has also been reported that the yield of mcl-PHAs from fatty acids such as nonanoic acid could achieve 0.66 to 0.69 g^−1^ mcl-PHA g nonanoic acid by co-feeding glucose [36]. Therefore, further optimization of the sequential-feeding process is needed to increase the yield of tailor-made mcl-PHAs.

## Conclusion

Introduction of *xylAB* from *E. coli* into *P. putida* KT2440 was sufficient to allow the recombinant to efficiently utilize xylose as the sole carbon source. Experiments performed in bioreactors showed that XylA and XylB were active in *P. putida* KT2440. The recombinant did not produce mcl-PHA from xylose, thus enabled production of a tailor-made mcl-PHA of up to 20% (w w^-1^) by sequential-feeding of xylose and octanoate. A maximal yield of 0.37 g mcl-PHA g^-1^ octanoic acid was obtained with PHA containing mainly 3-hydroxyoctanoate monomers (87% w w^-1^). Sequential feeding of relatively cheap carbohydrates and expensive fatty acids is a practical way to achieve more cost-effective mcl-PHA production. Optimization of initiation, rate and duration of feeding should be performed to achieve a higher yield and higher productivity of mcl-PHA. Furthermore, an optimized growth conditions will undoubtedly contribute to the economic feasibility of mcl-PHA production from renewable feedstock.

## Methods

### Bacterial strains and plasmids

Strains and plasmids used in this study are listed in Table 3.

**Table 3:** Strains and plasmids used in this study

**Strain or plasmid**	**Relevant characteristics**	**References**
*Strains*		
*P. putida* KT2440	Prototrophic, reference strain	http://openwetcare.org/wiki/E._coli_genotypes
*E. coli* W3110	Wild type, *xylAB* donor	http://openwetcare.org/wiki/E._coli_genotypes
*E. coli* JM109	*end*A1, *gln*V44, *gyr*A96, *thi*-1, *mcr*B^+^, *hsd*R17 (r_k_^–^, m_k_^+^), *rel*A1, *sup*E44, [F' *tra*D36 *pro*AB^+^*lac*I^q^*lac*ZΔM15]	http://openwetcare.org/wiki/E._coli_genotypes
*E. coli* HB101	F^-^, *hsd*S20 (r_B_^-^ m_B_^-^) *rec*A13, *ara*-14, *pro*A2, *lac*Y1, *gal*K2, *xyl*-5, *mtl*-1, *rps*L20 (Sm^R^).	http://openwetcare.org/wiki/E._coli_genotypes
*Plasmids*		
RK600	Cm^r^, ColE1, oriV, RK2, *mob+*, *tra+*	[38]
pVLT33	Km^r^, *Ptac*, MCS of pUCP18, hybrid broad-host-range expression vector	[39]
pSLM1	*xylA* and *xylB* cloned into pVLT33	this study

### Chemicals

All chemicals were purchased from Sigma-Aldrich (Buchs, Switzerland). The oligonucleotides were purchased from Microsynth (Balgach, Switzerland). The restriction enzymes were purchased from Fermantas GmbH (Nunningen, Switzerland) or New England Biolabs (Allschwil, Switzerland).

### Cloning, characterization and expression genes involved in xylose utilization

#### Construction of pSLM1

The chromosomal DNA of *E. coli* W3110 was extracted and used as the template for cloning of *xylAB* (GenElute™, bacterial Genomic DNA kit, Sigma-Aldrich). The fragment containing both *xylA* and *xylB* was amplified using the following primers: PFXylA (5’ CCGAATTCTGGAGTTCAATATG 3’) and PRXylB (5’ GATAAGCTTTACGCCATTAATG 3’). The amplified fragment was purified from agarose gel (GenElute^™^, Gel Extraction kit, Sigma Aldrich), and further digested with *Eco*RI and *Hin*dIII restriction enzymes. This digested fragment was ligated into the shuttle vector pVLT33 [39], which was cut with the same restriction enzymes. The ligation solution was transformed into *E. coli* JM109 and the recombinants were selected on a Luria broth agar plate with 50 μg mL^-1^ kanamycin, 1 M bromo-chloro-indolyl-galactopyranoside (X-gal), and 1 mM isopropyl β-D-1-thiogalactopyranoside (IPTG). The obtained plasmid (insert + vector) was named pSLM1. The nucleotide sequence of *xylAB* was analyzed and confirmed by GATC Biotech AG (Konstanz, Germany).

#### Introduction of pSLM1 into *P. putida* KT2440

The obtained plasmid pSLM1 was introduced into *P. putida* KT2440 by triparental mating [40]. *E. coli* HB101 (RK600) [38] was used as the helper strain. *E. coli* JM109 (pSLM1) was the donor strain and *P. putida* KT2440 was the acceptor strain. The *P. putida* KT2440 recombinants were selected on E2 medium (see below) containing 0.2% citrate and 25 μg mL^-1^ kanamycin. In analogy, the empty vector pVLT33 was also introduced into *P. putida* KT2440 as a control.

### Growth conditions

E2 minimal medium supplemented with different carbon sources (xylose, glucose or octanoic acid) was used throughout the whole study. This medium contains the following components: NaNH_4_HPO_4·_4H_2_O 3.5 g L^-1^, KH_2_PO_4_ 3.7 g L^-1^, K_2_HPO_4_ 7.5 g L^-1^, dissolved in water, and 1 mL L^-1^ MgSO_4·_7H_2_O 246.5 g L^-1^ and 1 mL L^-1^ of trace element (TE) dissolved in 1 M HCl were added. TE contains FeSO_4·_7H_2_O 2.78 g L^-1^, CaCl_2·_2H_2_O 1.47 g L^-1^, MnCl_2·_4H_2_O 1.98 g L^-1^, CoCl_2·_6H_2_O 2.38 g L^-1^, CuCl_2·_2H_2_O 0.17 g L^-1^, ZnSO_4__·_7H_2_O 0.29 g L^-1^. In some experiments, the nitrogen content of the E2 medium was reduced to 20% (0.2NE2) as indicated in the results section. If necessary, 25 μg mL^-1^ kanamycin was added to the culture medium.

#### Growth in shake flasks

The recombinant *P. putida* KT2440 (pSLM1) was pre-cultured in 100 mL of E2 medium containing 10 g L^-1^ xylose and 25 μg mL^-1^ kanamycin. The preculture in the exponential growth phase was then transferred to fresh E2 medium containing xylose and kanamycin with a dilution of 1:20. This transfer was repeated twice to allow cells to adapt to xylose. *P. putida* KT2440 (pVLT33) was used as control. The *P. putida* cells were grown at 30°C with an agitation of 150 rpm in 1 L baffled shake flasks.

*E. coli* cells were grown at 37°C in either LB medium or E2 medium containing xylose as the sole carbon source. Samples were taken and stored at −20°C as indicated in the Results section for the determination of xylose isomerase and xylulokinase activities.

#### Batch culture in 3.7 L reactor

The bioreactor study was carried out in a 3.7 L laboratory bioreactor (KLF 2000, Bioengineering, Wald, Switzerland) with a working volume of 2 L. Medium E2 supplemented with 10 g L^-1^ xylose as the sole carbon source was used. The batch bioreactor was inoculated with 300 mL of preculture having an OD_600_ of 2.10 and containing exactly the same medium as the one present in the bioreactor. The agitation was set at 750 rpm. The temperature was controlled at 30°C and the pH was maintained at 7.0 by automated addition of 4 M KOH or 2 M H_2_SO_4_. The dissolved oxygen tension was monitored continuously with an oxygen probe and kept at above 20% of air saturation (with a flow of 1 v v^-1^ min ^-1^).

#### Batch culture in 1 L mini-reactors

In order to obtain a nitrogen limited growth more rapidly, 0.2NE2 medium was used in these experiments. Four mini-reactor cultures (A, B, C and D) were grown in parallel in Multifors-Multiple benchtop bioreactors (Infors AG, Bottmingen, Switzerland). Temperature was controlled at 30°C and pH was maintained at 7.0 by automated addition of 4 M KOH or 2 M H_2_SO_4_. The dissolved oxygen tension was monitored continuously with an oxygen probe and kept at 30% of air saturation. Each reactor was inoculated using the pre-culture which was prepared as described above “Growth in shake flasks”. The initial OD_600_ in bioreactors was about 0.08. Kanamycin was added to a final concentration of 25 μg mL^-1^ when the recombinant was cultivated. Octanoic acid with different concentrations was fed to the bioreactors at different growth stages of the batches, as described in the Results section.

### Enzymatic assays

The cell pellets from batch culture were washed twice with 250 mM Tris–HCl buffer pH 7.5, and then lysed by the addition of lytic solution according to the manufacture’s instruction (CellLytic^™^ B Cell Lysis Reagent, Sigma-Aldrich, St. Louis, MO, USA). The samples were centrifuged at 20’000 g for 3 min in an Eppendorf centrifuge. The supernatant is referred as cell-free extract (CFE).

Xylose isomerase (EC 5.3.1.5) was measured in a solution containing 0.2 mM NADH, 50 mM xylose, 10 mM MgSO_4_, 0.5 U sorbitol dehydrogenase, and 30 μL CFE as it has been described previously [41]. The assay was performed in a 96-well plate at 30°C. The total volume of the assay was 200 μL. The consumption of NADH was measured spectrophotometrically at 340 nm using a plate reader (Bioteck Instruments GmbH, Luzern, Switzerland). One unit is defined as 1 μmole of consumed NADH min^-1^.

Xylulokinase (EC 2.7.1.17) was assayed as described previously [42]. The assay mixture contained 0.2 mM NADH, 50 mM Tris–HCl (pH 7.5), 2 mM MgCl_2_, 2 mM ATP, 0.2 mM phosphoenolpyruvate, 8.5 mM ATP, 2.5 U pyruvate kinase, 2.5 U lactate dehydrogenase and 30 μL CFE. The assay was performed at 30°C. The total volume was 200 μL in each well of the 96-well plate. The consumption of NADH was measured spectrophotometrically at 340 nm (Bioteck Instruments GmbH, Luzern, Switzerland). One unit is defined as 1 μmole of consumed NADH min^-1^.

### Analytical methods

#### Cell growth

Growth of bacterial cells was followed by measuring the optical density at 600 nm (OD_600_) using a UV-visible spectrophotometer (Genesys 6, ThermoSpectronic, Lausanne, Switzerland).

Cell dry weight (CDW) was determined using pre-weighed polycarbonate filters (pore size: 0.2 μm, Whatman, Scheicher & Schuell, Dassel, Germany). An appropriate volume (0.5 to 5 mL) of culture was filtered in order to obtain a biomass weight of about 2 mg per filter. The filter was dried overnight at 105°C, cooled down to room temperature in a desiccator and then weighted. The weight difference was used to determine the quantity of biomass per culture volume.

The optical density was correlated to cell dry weight with a ratio of CDW/OD = 0.6 when the cells did not contain PHA and with a ratio of CDW/OD = 0.5 when the cells contained PHA.

The maximum specific growth rate was calculated by the log of the biomass concentration (expressed in OD in this study) at time *t*_*2*_ minus the log of the biomass concentration at time *t*_*1*_ divided by the time interval during the exponential growth phase.

(none) $$\mu =\frac{\ln X_{2}-\ln X_{1}}{t_{2}-t_{1}}$$

X = biomass concentration expressed in OD values

#### Measurement of carbon sources

The consumption of carbon sources was measured by HPLC-MS. Samples were diluted to 0.01 to 0.1 mM with 50% acetic acid and 50% acetonitrile (v v^-1^) and loaded on a reversed phase C18 column (Gemini C18 5 micron, 250 mm x 4.60 mm, Phenomenex, U.K.). A gradient of 100% diluted formic acid (0.1 v % in water) to 100% acetonitrile was applied as the mobile phase. The flow rate was 0.8 mL min^-1^ and the gradient was completed after 25 min. The peaks were detected by electrospray ionization (ESI) in negative mode [43]. The standard curves for xylose and octanoic acid were recorded in the range of 0.01 to 1 g L^-1^, and 0.005 g L^-1^ to 0.03 g L^-1^, respectively.

#### Ammonium concentration

NH_4_^+^-nitrogen consumption was detected using an ammonium test kit following the manufacturer instruction (Merck KGaA, 64271 Darmstadt, Germany). The detection limit was 0.01 NH_4_^+^-N mg L^-1^. The method was linear up to 3.0 mg N L^-1^, above which dilution with distilled water was needed.

#### Acetic acid measurement

A DX-500 ion chromatography system (Dionex, Sunnyvale, CA, USA) was used to analyze the acetic acid production during the co-feeding experiments. IonPac AS 11 HC (250 mm × 4 mm) and AG 11 HC guard (50 mm × 4 mm) columns were used. Sodium hydroxide gradient of 0.5 to 30 mM allowed the identification and the quantification of this organic acid in 15 min.

#### PHA content

For analysis of intracellular PHA the culture broth was centrifuged (10,000 x g; 4°C; 15 min) and the cell pellet was lyophilized for 48 h. Pyrex vials were weighed to determine the exact transferred biomass, then 2 mL of 15% v v^-1^ H_2_SO_4_ in methanol were added. Furthermore, 2 mL of methylene chloride containing 2-ethyl-2-hydroxybutyrate (10 g L^-1^) were added as internal standard. The suspension was boiled at 100°C for 2.5 h in an oven. The samples were cooled on ice; then 1 mL of distilled water was added in order to extract the cell debris that is soluble in the aqueous phase. The sample was mixed by vortexing for 1 min. The complete water phase was discarded (upper phase), including droplets hanging on the tube wall and including the top layer of the chloroform phase. Na_2_SO_4_ powder was added to dry the methylene chloride phase. Two hundred μL of the chloroform phase was filtered using solvent resistant filters (PTFE, 0.45 μm) and transferred to a GC sample tube. PHA content and monomer composition were subsequently analyzed on a GC (A200s, Trace GC 2000 series, Fisons Instruments, Rodano, Italy) equipped with a polar fused silica capillary column (Supelcowax-10: length 30 m; inside diameter 0.31 mm; film thickness 0.5 μm; Supelco, Buchs, Switzerland). Helium was used as carrier gaz (3 mL min^-1^) and detection was performed with a flame ionization detector (FID) at 285°C. The temperature was increased from 10°C to 280°C at a rate of 10°C min^-1^.

##### Reproducibility

All measurements for growth and PHA assays were performed at least in duplicates. The measurements for XylAB assays were performed in triplicates. The data presented in this report are the average numbers.

## Competing interests

The authors declare that they have no competing interests.

## Authors’ contributions

SLM carried out the experiments, and drafted the manuscript. MZ and TE participated in the design of the study and helped to draft the manuscript. LTM helped with manuscript preparation. QR conceived of the study, and participated in its design and coordination and helped to draft the manuscript. All authors read and approved the final manuscript.
